# The First Report on the Administration of the Act

**Published:** 1913-07-26

**Authors:** 


### NATIONAL INSURANCE ACT DEPARTMENT.

### LXII.—The First Report on the Administration of the Act.

When in the future a historian essays to write the history of National Health Insurance in the United Kingdom his first step, we imagine, will be to master the contents of the Report recently issued by the Insurance Commissioners. The report is so full, and yet so concise, that it becomes a really difficult matter to give an adequate idea of its contents in the limited space we are able to devote to its consideration.

The volume impresses one first by its bulk, and its great size must inevitably deter many persons from commencing to read what would at first sight demand so much attention. A closer acquaintance with the Report, however, leads to the conclusion that the time spent in mastering its contents is by no means wasted.

From the prefatory memorandum above the name of Mr. C. F. G. Masterman we gather that the Report is put forward as a full description of the administration of Part I. of the National Insurance Act, and that the wealth of detail, which is so largely responsible for the bulk of the volume, is furnished in order that those who are interested in Health Insurance, whether in the United Kingdom or in other countries, may have a permanent record of the difficulties which have been encountered and the means which have been adopted to overcome them.

The Report is itself a monumental work, showing on every page clear evidence of the pains that have been taken in its compilation; but it chronicles a far greater achievement, which can now be claimed to have been accomplished, in the initiation of the system of National Health Insurance in the United Kingdom.

The average Briton has a short memory, and so used have we already become to the requirements of National Insurance that it may come as a surprise to some to recollect that it is little more than eighteen months since the National Insurance Act became law. It is shown in the Report that in that incredibly short space of time marvels of administration have been accomplished far beyond the conception of any politician when the scheme was in contemplation, and so stupendous as to make us wonder, on looking backwards, how it has all been done in so short a period.

While much of the volume is necessarily concerned with setting forth matters that were common knowledge at the time of their happening, it is both interesting and instructive to look back on the progress that was made step by step in carrying the scheme into operation. This retrospect is interesting because each stage of development had its own special problems and difficulties, and instructive because the experience gained and recorded should enable many difficulties to be foreseen—and thus avoided—in the future development of the present complex scheme.

### A Gigantic Enterprise.

The gigantic proportions of the enterprise cannot be put more forcibly than in the words of Mr. Masterman when he states that: "The Report includes an account of the formation of an Insurance Fund of nearly twenty million pounds; the bringing into insurance of nearly fourteen million persons; the constitution and work of 236 Insurance Committees. . . ."

The contents of the volume are divided into six parts, the first of which is in the nature of an introduction and gives in four pages the best brief outline of the provisions and scope of the Act which we remember to have seen. Part II. deals with the work of the Joint Committee of the Insurance Commissioners, and Parts III., IV., V., and VI. with that of the English, Scottish, Irish, and Welsh Commissioners respectively. Appendices follow in which reports of various Committees and statistical and other tabular matter are furnished.

### The Joint Committee and the Supply of Information.

The Joint Committee was appointed in accordance with Section 83 of the Act for the specific purpose of co-ordinating the efforts of the several Commissions in all matters where uniformity was desirable. Thus it was the Joint Committee which was responsible for all actuarial matters—*e.g.*, those involving the calculation of rates of contribution, reserve values, transfer values, and the like—and for the appointment of Special Committees to consider the position of certain special classes under the Act.

The principal interest in the work of the Joint Committee which is contained in the Report is, however, in regard to the steps taken to supply information concerning the Act to the enormous population whom it concerned. This was done by means of specially trained lecturers and by the use of leaflets. In England alone seventy-four lecturers were appointed, and when their engagements terminated they had attended over 1,700 meetings and addressed 900,000 people.

The issue of leaflets was even more remarkable, for we are informed that the total number circulated was over 33 millions, in addition to the distribution of the National Health Insurance Leaflet, a copy of which was delivered through the Post Office to every householder in the United Kingdom.

These measures had the effect, when combined with the efforts of the approved societies, of leaving less than 4 per cent. of the insured persons outside these societies.

### The Approval of Societies.

While the Joint Committee was thus busily engaged with matters relating to the general dissemination of information the several Insurance Commissions were taking active steps to obtain the necessary machinery in the form of approved societies through which the benefits of the Act were to be administered. The account given of the operations of the English Commissioners to this end is particularly interesting. At first the smaller societies held back from seeking approval, apparently on account of the misapprehension that a minimum membership of 5,000 would be required. When this misconception was removed applications were made from all classes of previously existing registered and unregistered friendly societies, trade unions, etc., so that ultimately over 2,000 societies obtained approval. This does not, however, give an adequate idea of the extent of the administrative machinery, for a society with branches is approved as a single society and the branches do not obtain approval separately. There are altogether over 20,000 branches of duly approved societies, and when it is remembered that the societies formed in connection with the industrial assurance offices and collecting friendly societies have over 60,000 district representatives or agents, it is clear that the Act has called into being a vast army for its administration.

Special emphasis is laid in the Report on the measures adopted to enable small societies to obtain approval and on the advantages which have accrued through the formation of associations of such societies.

### Maternity Benefit—Midwives' Institute's Scheme.

In one of the paragraphs dealing with matters of special difficulty (Par. 231) the English Commissioners call attention to a scheme which has been put into operation by the Midwives' Institute. "The scheme provides for a guarantee against the risk of the prescribed fee having to be deducted from maternity benefit. Any midwife who is a member of the Institute can, under the scheme, increase her ordinary fee for attendance at a confinement by one shilling, which is paid into a common fund, and out of this fund, which is under the control of the Institute, the prescribed fee will be paid in the event of a doctor having to be summoned. Any deduction from the maternity benefit for the purpose of paying the prescribed fee would thus be avoided."

We were not previously aware of this scheme for overcoming the difficulty imposed upon approved societies in regard to the payment of maternity benefit when the patient elects to be attended by a midwife. It is, as will be seen, a form of private insurance whereby the insured person by paying 1s. will obtain the advantage of receiving the maternity benefit immediately after the confinement, instead of possibly having to wait a fortnight, and will receive the full 30s. in any case without the possibility of the deduction of the doctor's fee should it be necessary for a doctor to be called in.

Until such time as Section 18 of the Act has been amended, this scheme would appear to have much to commend it. Other schemes relating to the administration of maternity benefit otherwise than in cash for the benefit of the mother are also described—the chief difficulty which has prevented the adoption of such schemes is the necessity of preserving to the mother, under the terms of the Act, the right of selecting the doctor or midwife by whom she wishes to be attended.

### Sickness and Maternity Benefits in Operation.

The Commissioners conclude their remarks on the administration of sickness and maternity benefits by the approved societies with the statement that "they are satisfied that, taking into account the difficulties necessarily arising in putting so large a scheme into operation, the scrutiny of claims and payment of benefit has in general been efficiently and promptly carried out. The causes of what delay has occurred in dealing with claims vary as between small and large societies. The small societies often required longer to become familiar with the bearing of their State rules upon particular claims, and a claimant whose case presented any unusual feature was at first liable to have payment of his benefit delayed. The large centralised societies have been apt to find their office organisation inadequate for dealing with the first batch of claims, and have therefore been unable to give prompt advice to their local agents. These defects are being removed in the smaller societies principally by the progress of time, in the larger societies by improved organisation. At the same time this increased efficiency in the approved societies is being supplemented and encouraged by the growing familiarity of insured persons themselves with their position and rights under the Act."

Note.—Questions and Correspondence on all doubtful points are invited, and should be addressed to the Editor, The Hospital, 28, 29 Southampton Street, Strand, London, W.C., and marked "Insurance."

